# Environmental and sociodemographic factors associated with household malaria burden in the Congo

**DOI:** 10.1186/s12936-019-2679-0

**Published:** 2019-02-26

**Authors:** Nlandu Roger Ngatu, Sakiko Kanbara, Andre Renzaho, Roger Wumba, Etongola P. Mbelambela, Sifa M. J. Muchanga, Basilua Andre Muzembo, Ngombe Leon-Kabamba, Choomplang Nattadech, Tomoko Suzuki, Numbi Oscar-Luboya, Koji Wada, Mitsunori Ikeda, Sayumi Nojima, Tomohiko Sugishita, Shunya Ikeda

**Affiliations:** 1 School of Medicine and Graduate School of Public Health, International University of Health and Welfare, Narita, Japan; 2 Graduate School of Nursing, University of Kochi, Kochi, Japan; 3 Western Sydney University, Perth, Australia; 4 Faculty of Medicine, University of Kinshasa, Kinshasa, Democratic Republic of Congo; 5 Department of Environmental Medicine, Kochi University Medical School, Nankoku, Japan; 6 National Center for Global Health and Medicine, Tokyo, Japan; 7 Department of Public Health, University of Kamina, Kamina, Democratic Republic of the Congo; 8 School of Public Health, University of Lubumbashi, Lubumbashi, Democratic Republic of the Congo; 9 Tokyo Women’s Medical University, Tokyo, Japan

**Keywords:** Environment, Household malaria, Income, Sanitation

## Abstract

**Background:**

Malaria is one of the most severe public health issues that result in massive morbidity and mortality in most countries of the sub-Saharan Africa (SSA). This study aimed to determine the scope of household, accessibility to malaria care and factors associated with household malaria in the Democratic Republic of Congo (DRC).

**Methods:**

This was a community-based cross-sectional study conducted in an urban and a rural sites in which 152 households participated, including 82 urban and 70 rural households (1029 members in total). The ‘malaria indicator questionnaire’ (MIQ) was anonymously answered by household heads (respondents), reporting on malaria status of household members in the last 12 months.

**Results:**

There were 67.8% of households using insecticide-treated bed nets (ITN) only, 14.0% used indoor residual spraying (IRS) only, 7.3% used ordinary bed nets (without insecticide treatment), 1.4% used mosquito repelling cream, 2.2% combined ITN and IRS, whereas 7.3% of households did not employ any preventive measure; p < 0.01). In addition, 96.7% of households were affected by malaria (at least one malaria case), and malaria frequency per household was relatively high (mean: 4.5 ± 3.1 cases reported) in the last 12 months. The mean individual malaria care expenditure was relatively high (101.6 ± 10.6 USD) in the previous 12 months; however, the majority of households (74.5%) earned less than 50 USD monthly. In addition, of the responders who suffered from malaria, 24.1% did not have access to malaria care at a health setting. Furthermore, a multivariate analysis with adjustment for age, education level and occupation showed that household size (OR = 1.43 ± 0.13; 95% CI 1.18–1.73; p < 0.001), inappropriate water source (OR = 2.41 ± 0.18; 95% CI 1.17–2.96; p < 0.05) absence of periodic water, sanitation and hygiene (WASH) intervention in residential area (OR = 1.63 ± 1.15; 95% CI 1.10–2.54; p < 0.05), and rural residence (OR = 4.52 ± 2.47; 95% CI 1.54–13.21; p < 0.01) were associated with household malaria.

**Conclusion:**

This study showed that household size, income, WASH status and rural site were malaria-associated factors. Scaling up malaria prevention through improving WASH status in the residential environment may contribute to reducing the disease burden.

## Background

Recent reports from the World Health Organization (WHO) show progress made by several countries in reducing malaria burden, with a decline in preventable child deaths over the 15-year period of the implementation of millennium development goals (MDGs). Epidemiologic figures show that over 6.2 million malaria deaths were averted between 2000 and 2015 in sub-Saharan Africa (SSA) [1]. Malaria is an infectious disease that continues to pose a major health challenge; it is still endemic in 97 countries, including the Democratic Republic of Congo (DRC) where only 56% of children under age five sleep under insecticide-treated bed nets [2–4]. In several malaria-affected countries, scale-up interventions are being implemented to reduce the disease burden, such as long-lasting insecticide-treated nets (LLIN), indoor residual spraying (IRS), rapid diagnostic tests (RDT) and artemisinin-based combination treatment (ACT).

Despite the progress made in the fight against malaria, a number of challenges are currently reducing the impact of antimalarial interventions such as the emergence of drug-resistant malaria parasites and mosquitoes resistant to insecticides, and the existence of fake malaria drugs [3, 5, 6]. Furthermore, many of high malaria burden countries such as Nigeria and DRC have been reporting significant increases in disease cases [4], suggesting the necessity for further research that could culminate in the discovery of new strategies for malaria control.

Primary health care (PHC) system has been playing a crucial role in reducing malaria burden in endemic countries, and such an achievement can only be reached if adequate infrastructure, delivery mechanisms and uptake for PHC coverage are available, in addition to minimum acceptable income. Moreover, in the context of malaria endemic countries, there is still a need to make malaria preventive and curative treatments not only available but also accessible [7–9]. In DRC, given extreme poverty and the dysfunction of the national health system due to cyclic armed conflicts, insecurity and governance issues, universal health coverage (UHC) remains a dream. In DRC, poverty-related inaccessibility to health care has caused increased rates of self-medication and use of herbal medicines [10, 11].

This first ‘Congo Malaria and Environment study’ was conducted to determine the frequency of malaria in households, malaria care accessibility and factors associated with household malaria, as well as the impact of water, sanitation, hygiene (WASH) status and income on disease burden.

## Methods

### Study design, sites and participants

This was an observational community-based cross-sectional study conducted in two sites located in two different provinces: Kasangulu, a rural town in Kongo central province, and Limete, an urban county located in the capital Kinshasa. Invitations were sent to two communities in each study sites to join this survey and 181 survey sheets were distributed, of which 152 were completed. Thus, this study sample was 152 households (82 from urban and 70 from rural study sites), consisting of 1029 individuals. Home visits were carried out for to evaluate WASH status, the quality of malaria prevention tools used by households.

Participation was voluntary, and heads of households were enrolled in their respective communities (cultural or social centre, club and church) after attending meetings in which a thorough explanation about the survey was delivered. Each participating community provided a codified list of volunteer participants. The same codes were transcribed on questionnaire sheets that were later distributed to participants.

### Survey questionnaire

The ‘Malaria Indicator Questionnaire (MIQ) [12, 13], a standardized structured and self-administered survey questionnaire, was used in this study. MIQ is a composite questionnaire that comprises items that correspond to five main themes, including personal and household sociodemographic and clinical characteristics of respondents (household heads) at the time of this survey, lifestyle pattern, personal and family medical history, and information on WASH status at home and in the living environment, and malaria preventive measures and care options. It is a survey questionnaire from ‘The World Bank and WHO Malaria Programme’ in Africa, commonly used by many western and eastern African countries to evaluate their respective malaria programmes. Considering the relatively high illiteracy rate in the country, the questionnaire was self-administered for educated respondents; whereas, for illiterate respondents, the team of surveyors (which included trained community health nurses and a medical doctor) explained each question in a local dialect and guided them.

### Study outcome variables

For this study, the outcomes were household malaria incidence (number of incident cases) in the previous 12 months, and accessibility to malaria care at a medical settings.

### Ethical considerations and data analysis

Ethical approvals of the study were obtained from the ethics committee of the Graduate School of Nursing, University of Kochi, Japan, and the School of Public Health of the University of Lubumbashi, DRC. Signed written informed consent was obtained from each household head.

For data analysis, Stata statistical software version 14 (Stata Corp.; TX, US) was employed. Differences between categories of qualitative variables (study site, sociodemographic characteristics, preventive measures, and place of malaria care) and dichotomized variables (WASH parameters, range of household malaria cases, and household income status) were assessed with the use of Chi square test. The breakdown of WASH components was performed as follows:

1. Household latrine (toilet): appropriate (pit latrine with slab and flush; pit latrine with slab and manual flush) or inappropriate (pit latrine without slab);
2. Household water source: appropriate (indoor tap water) or inappropriate (outdoor tap water or water well).

Variables that showed significant and marginally significant associations with household malaria in the bivariate model were entered into the multivariate logistic regression model to determine the factors associated with malaria. P-values (double sided) less than 0.05 were considered significant.

## Results

### Characteristics of the households and the respondents

Of the 181 survey questionnaires distributed to household heads (responders), 152 (83.9%) were returned; 51.3% of respondents were males and 48.7% females. Of the responders, 40.2% were aged 30 to 39 years, and 65.1% were married. Additionally, the majority (78.3%) of respondents were unemployed. Regarding educational level of the respondents, a greater proportion (41.5%) had high school level (Table 1). On one hand, the majority (54%) of households were from urban area, had a larger size (53.3%; range: 6–18 members), had a monthly income less than 50 USD (75%), used either water well or outdoor tap water (58.3%), and used pit latrine without slab (uncovered toilet, 78.2%); on the other hand, 37.5% of households shared latrines with neighbors, 82.9% owned a radio, but only 58.6% owned a television (TV) set as shown in Table 1.

**Table 1:** Characteristics of respondents and households

Characteristics	N	%
(1) Respondents (household heads; n = 152)		
Gender		
Male	78	51.3
Female	74	48.7
Age (years)		
20–29	24	15.8
30–39	61	40.2
40–49	37	24.3
50–70	30	19.7
Marital status		
Married	99	65.1
Divorced	6	4.0
Widowed	8	5.2
Single with children	22	14.5
Single without children	17	11.2
Education		
Never gone to school	11	7.2
Primary	8	5.3
High school	63	41.5
Technical school	25	16.4
University/College	45	29.6
Occupation		
Unemployed	119	78.3
Employed	33	21.7
(2) Households (total members: n = 1029)		
Family/household size		
2–5	71	46.7
6–18	81	53.3
Households with children < 5 years		
No	45	29.6
Yes	107	70.4
Residential area		
Rural	70	46.0
Urban	82	54.0
Monthly income status		
Overall less than 50 USD	114	75
50 USD or more	38	25
Rural less than 50 USD	62	88.6
50 USD or more	8	11.4
Urban Less than 50 USD	48	58.5
50 USD or more	34	41.5
Access to electricity		
Yes	134	88.2
No	12	7.9
No answer	6	3.9
Use of means of communication		
Television (yes)	89	58.6
Radio (yes)	126	82.9
Telephone (yes)	133	87.5
Type of toilet used (n = 147)		
Pit latrine with slab and flush	10	6.8
Pit latrine with slab/manual flush	22	14.9
Pit latrine without slab	115	78.2
Shared latrine with neighbors (n = 152)		
Yes	88	57.9
No	57	37.5
Other	7	4.6
Water source (n = 151)		
Indoor tap water (yes)	63	41.7
Outdoor tap water or water well (yes)	88	58.3

*USD* United States’ dollar

**Fig. 1:**
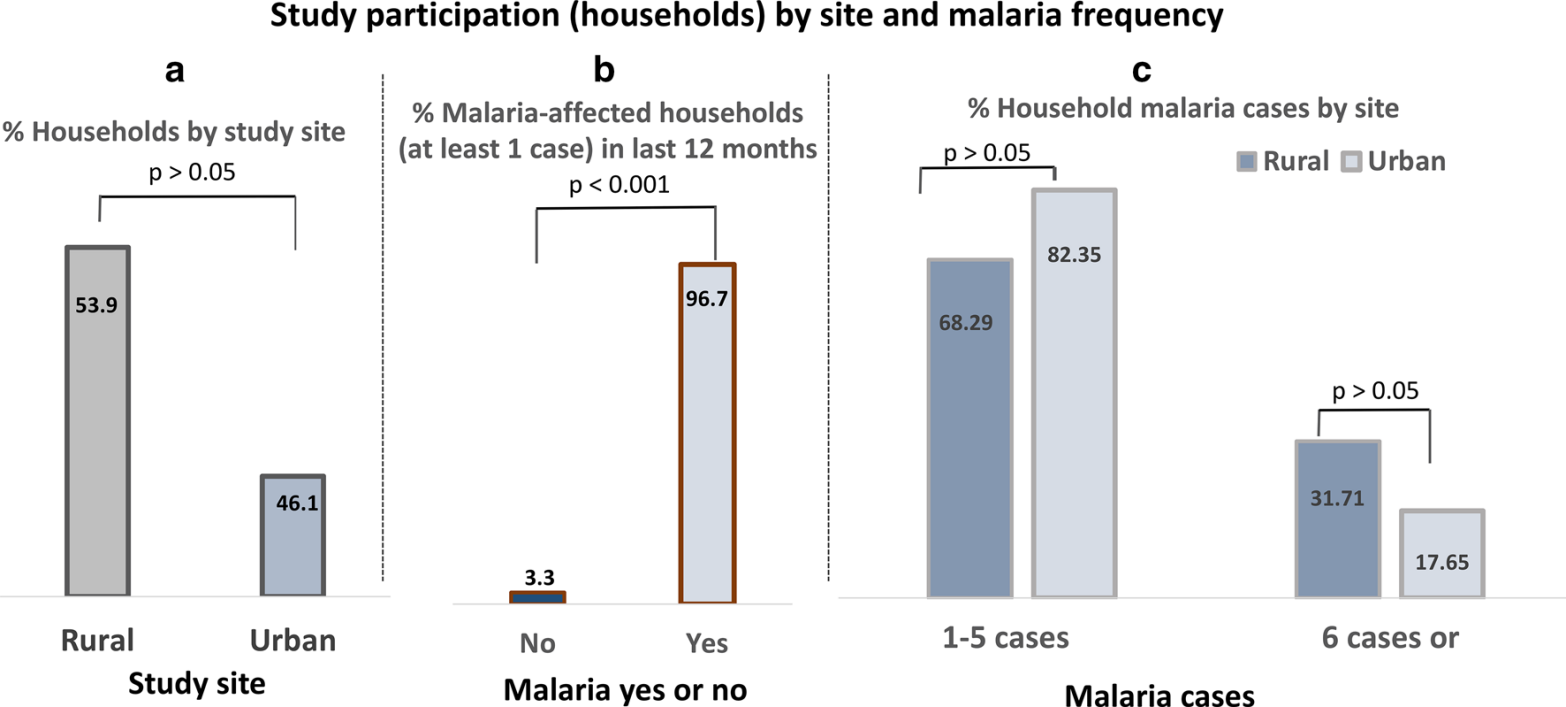
Proportion of households (**a**), proportion of malaria-affected households (**b**) and malaria frequency (**c**) by study site. The figure shows that there was no significant difference between households from rural and urban sites in terms of participation rate and frequency of malaria in the previous 12 months, whereas a markedly higher proportion of households (96.7%) reported at least one malaria case in the last 12 months (vs. 3.3% for non-affected households; p < 0.001)

Trend of household malaria frequency in the last 12 months according to study site, preventive measures used and income status. As shown in Fig. 1, a relatively higher proportion (53.9%) of households were from the rural study site (vs. 46.1% from urban site; p > 0.05) (Fig. 1a). The overall proportion of malaria-affected households (at least one malaria case) was high (96.7%) in the last 12 months (Fig. 1b); a relatively higher proportion of rural households reported a higher malaria frequency (6 cases or more) in the last 12 months, as compared with urban households (31.7% vs. 17.6%; p > 0.05), but not significantly (Fig. 1c).

**Fig. 2:**
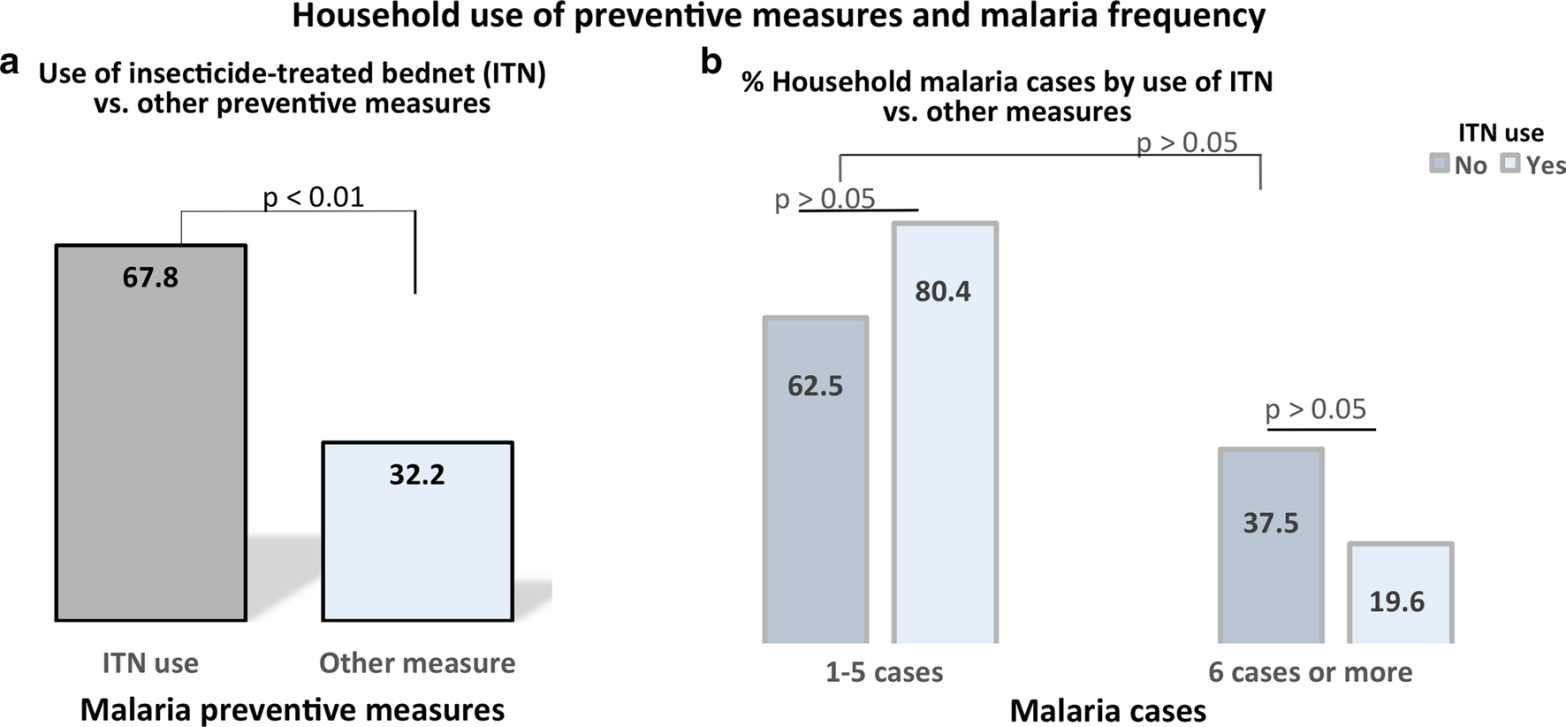
Household use of preventive measures (**a**) and malaria frequency (**b**). The figure shows that the majority of households owned insecticide-treated bed net (ITN); however, malaria frequency was not significantly different among households when considering the use of preventive measures (ITN users vs. ITN non-users). ITN: insecticide-treated bed nets; p, p-value

**Fig. 3:**
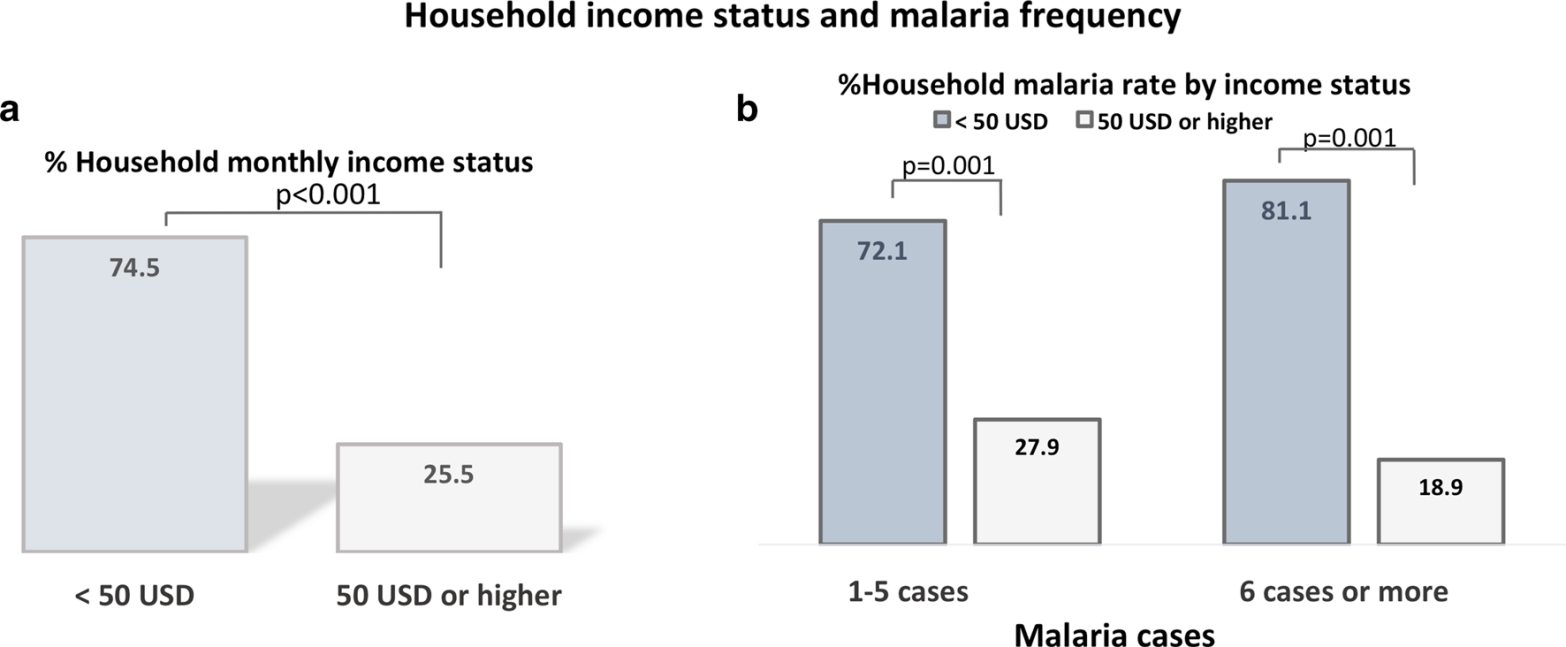
Household income status (**a**) and malaria frequency (**b**). The figure shows that a higher proportion of households earning less than 50 USD monthly (**a**), and that poorer households tended to have higher frequency of malaria (**b**). USD: American dollar; p: p-value

The rate of use of insecticide-treated bed nets (ITN) among participating households was also assessed, and it was observed that the majority of households (67.8%) used ITN. Of the remaining 32.2%, 14.0% used indoor residual spraying (IRS), 2.2% combined ITN and IRS, 7.3% used ordinary bed nets (without insecticide), and 1.4% used mosquito repelling cream (p < 0.01) (Fig. 2a). In addition, no significant difference was observed when comparing the proportion of malaria-affected ITN users with ITN non-users (Fig. 2b). Regarding monthly income status, a higher (74.5%) proportion of households earned less than 50 USD (vs. 25.5% earning 50–700 USD; p < 0.001), and malaria occurred more frequently in households earning less than 50 USD as compared with those earning more (p < 0.01) (Fig. 3).

### Trend of household malaria frequency in the previous 12 months according to sanitation (WASH) status in the residential area

**Fig. 4:**
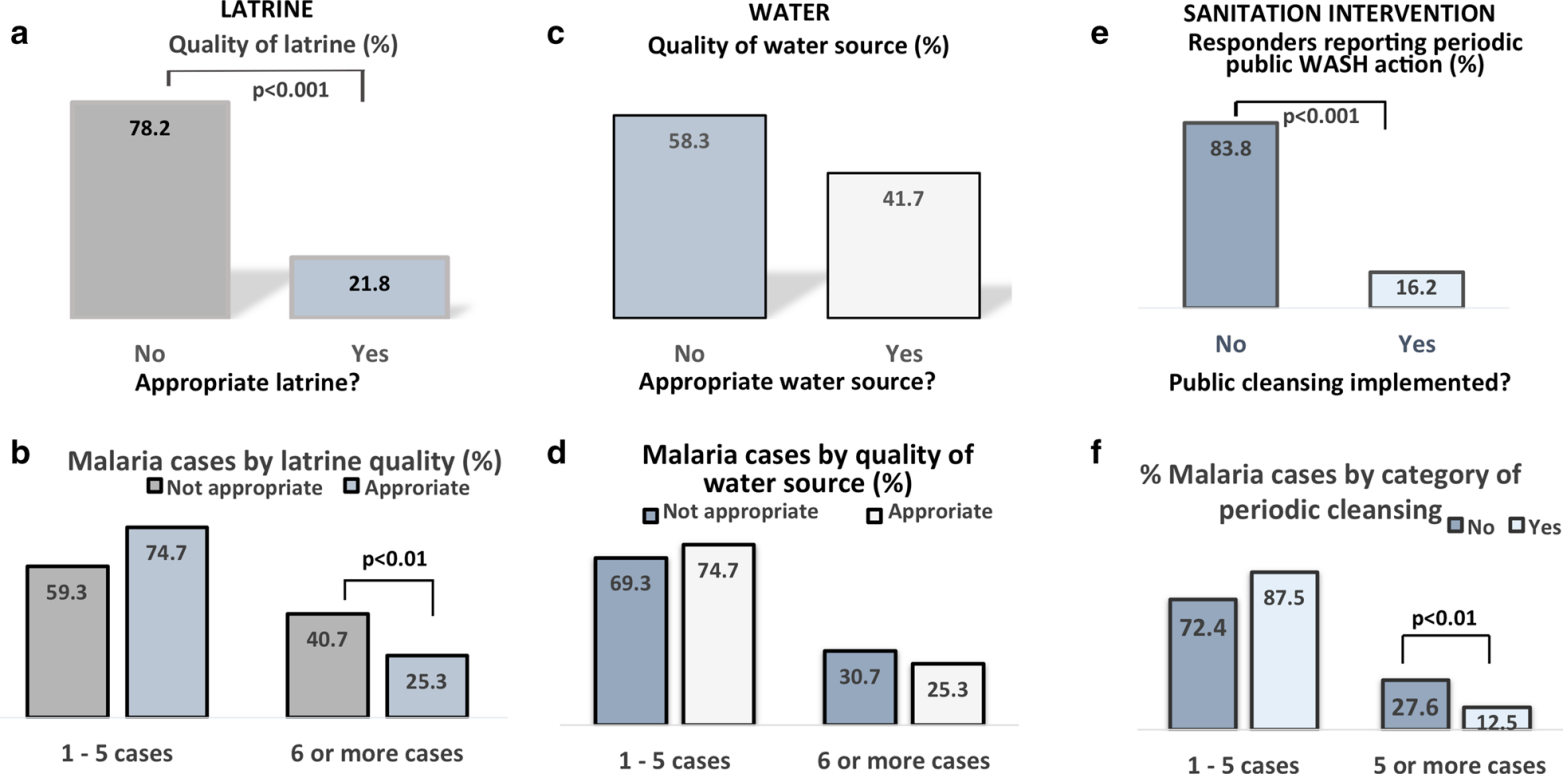
Malaria frequency by quality of latrine (**a**, **b**), quality of water source (**c**, **d**) and presence/absence of periodic sanitation intervention (**e**, **f**). The figure shows significantly higher frequency of malaria in households sing inappropriate latrine (**b**) and those living in areas with absence of periodic sanitation intervention (**f**)

Mean number of household malaria cases reported by the respondents was relatively high, 4.5 ± 3.1. Figure 3 shows the status of water, sanitation and hygiene (WASH) in the living environment. It was observed that a significantly higher (78.2%) proportion of households used inappropriate latrines (vs. 21.8% using appropriate latrines; p < 0.001) (Fig. 4a); and a significantly higher proportion of households using inappropriate latrine (40.7%) had a high malaria frequency (6 cases or more) in the last 12 months (vs. 25.3% of household using appropriate latrine; p < 0.01) (Fig. 4b). Additionally, there were 58.3% of households used inappropriate water source (vs. 41.7% for households using appropriate one) (Fig. 4c); however, no significant difference in malaria rate was observed when comparing households in term of quality of water source (Fig. 4d).

Furthermore, regarding the sanitation actions in the living environment, a higher (83.8%) proportion of respondents reported the absence of implementation of periodic WASH intervention in their residential area (vs. 16.2% of those reporting such an intervention; p < 0.0001) (Fig. 4e). Consequently, households living in areas without WASH action were more likely to report malaria cases (vs. households from areas with periodic WASH intervention; p < 0.001) (Fig. 4f).

### Malaria care accessibility and settings for infected respondents

**Fig. 5:**
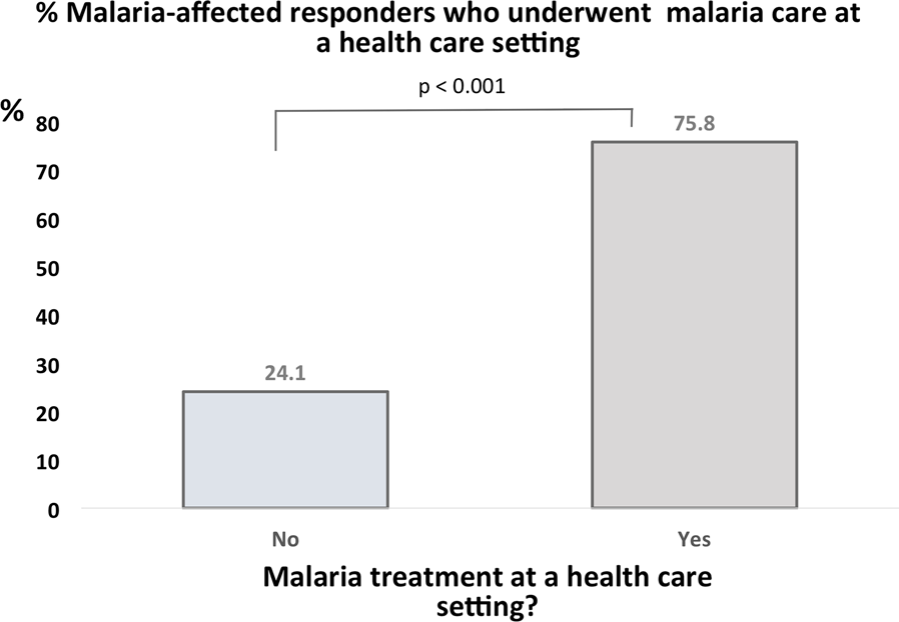
Accessibility to malaria care by affected respondents. The figure shows that 24.1% of malaria-affected respondents did not undergo malaria treatment at a health setting. Instead, they used either a traditional remedy or self-medication

In this study, 90.1% (137/152) of the respondents who reported having developed at least one malaria episode in the previous 12 months were asked to mention whether malaria care was provided at a health setting (hospital, health centre or dispensary) or whether they opted for self-medication. Of them, 75.8% were taken care at a health setting, and the remaining 24.1% opted either for self-medication (17.8%) or the use of a traditional malaria therapy (6.3%) after getting tested positive for malaria at a local health setting (Fig. 5).

### Association between household sociodemographic characteristics, WASH status and household malaria by logistic regression analysis

A bivariate and multivariate logistic regression analyses were performed to determine factors associated with household malaria. In the bivariate analysis, the following factors were significantly associated with household malaria: household size (OR = 1.30 ± 0.08; 95% CI 1.14–1.49; p < 0.001), low household income (OR = 1.71 ± 0.28; 95% CI 1.23–3.51; p < 0.05), and periodic WASH intervention (OR = 1.86 ± 0.23; 95% CI 0.10–1.26; p < 0.05). After adjusting for education, age and occupation, the multivariate analysis showed that household size (OR = 1.43 ± 0.13; 95% CI 1.18–1.73; p < 0.001), inappropriate water source (OR = 2.41 ± 0.18; 95% CI 1.17–2.96; p < 0.05), periodic WASH intervention (OR = 1.63 ± 1.15; 95% CI 1.10–2.54; p < 0.05), and rural residence (OR = 4.52 ± 2.47; 95% CI 1.54–13.21; p < 0.01) were significantly associated with household malaria. No significant association was observed between household malaria, ITN use, and type of toilet, as shown in Table 2.

**Table 2:** Association between household sociodemographic characteristics, WASH status and household malaria by multivariate logistic regression analysis

Variable	OR	SE	95% CI	P	aOR	SE	95% CI	Adjusted P
Household size	1.3	0.08	1.14–1.49	*< 0.001*	*1.43*	0.13	1.18–1.73	*<* *0.001*
Preventive measures (ITN vs. other)	0.41	0.15	0.18–0.87	*0.053*	0.47	0.23	0.17–1.25	0.133
Periodic WASH intervention (Yes vs. no)	1.86	0.23	0.10–1.26	*<* *0.05*	1.83	1.15	1.10–2.54	*<* *0.05*
Water source (appropriate?: no vs. yes)	0.48	0.19	0.22–1.07	0.076	*2.41*	0.18	1.17–2.96	*<* *0.05*
Monthly income (< 50 USD vs. 50–700 USD)	1.71	0.28	1.23–3.51	*<* *0.05*	1.68	0.45	1.46–2.36	*<* *0.05*

*ITN* Insecticide-treated bed net, *aOR* adjusted odds ratio, *p* p-value, *WASH* water-sanitation-hygiene status, *USD* United States’ dollar

Italic values indicate the significance of* p*-values less than 0.05

## Discussion

The present study aimed to determine factors associated with malaria on household level in a sample of 152 Congolese households (1029 household members) from two counties (one in the capital Kinshasa and another one in the rural area of Kongo Central province). This study also sought to determine sociodemographic and environmental factors associated with malaria.

Findings showed that respondents from households with lower monthly income, low literacy and those who reported the absence periodic WASH intervention in the residential area were more likely than others to report malaria. A recent study carried out among South African women also showed that low literacy and low household income were associated with a history of malaria [14]. On one hand, regarding the relationship between malaria and economic status, the disease is known to cause or deepen poverty through spending on health care and loss of income, and people living in extreme poverty such as in rural DRC are at a high risk of being infected, given the difficulty to have access to effective preventive measures and malaria care [15–17]. On the other hand, the study did not show any association between education and malaria. One possible reason could be the hyperendemic status of malaria in districts where the study was conducted, given the proximity to river and numerous stagnant water spots during the rainy season, with a huge mosquito population as a consequence. Future studies will investigate the contribution of these factors in malaria prevalence in DRC.

Access to health care services is considered crucial to improving the health of populations [7]. In several developing countries, PHC has rendered malaria diagnosis and care affordable even to people living in remote areas in Africa [18]. However, in DRC, a country with high rate of unemployment, inexistence of national health insurance system and where the majority of the population live in extreme poverty, malaria diagnosis and care are not affordable to many households. In fact, for almost three decades, the Congolese government and most private companies pay to employees monthly allowances different from salaries decided by the country’s law. For instance, in the health sector, the monthly income for a nurse approximately 101 USD, and 122 USD for a laboratory technician [19]. With the current Congo crises, the country’s economy has worsen with a record currency debasement and hyperinflation; some public servants’ monthly allowance is actually less than 50 USD. According to USAID, large segments of the Congolese population currently live below the poverty line threshold of 1.25 USD per day [20].

This study showed that 24.1% of malaria-affected respondents did not get treated at a health setting when they were infected, even when the diagnosis was made in a medical setting. In fact, self-medication is quite a common behavior in DRC for poor households, as for some other countries of the sub-Saharan region [21, 22]. When an individual is diagnosed with malaria, but cannot afford the treatment cost, he or she may purchase malaria drug a local a drugstore, sometimes ignoring the possible consequences of this practice.

This study showed higher frequency of malaria in households from the rural site in the previous 12 month-period, as compared with urban ones. Similar trends were found in other reports from SSA countries. For example, a study conducted in the northern Zambian district of Nchelenge between 2012 and 2015 in 2486 individuals from 742 households showed a malaria rapid diagnostic test (RDT) positivity that reached 52% in 2015 [23]. Another study by Ajani and Ashigidigbi [24] conducted in a Nigerian rural district found that low level of awareness and large household size were among the major risk factors for malaria in rural households.

Furthermore, vector control through ITN and IRS is one of the measures that are reported to be effective as they reduce mosquito population and bites and, in DRC, the use of ITN is the main preventive measure that is promoted, given that not all households can afford the cost of IRS and mosquito repelling skin lotion. This study showed that the majority of households used ITN. Although a relatively higher proportion of household using ITN were found to have lower malaria frequency as compared with the group of those who did not use ITN, no association was observed between ITN use and household malaria.

There are previous studies that reported association between ITN use and malaria incidence [25, 26]. This fact can be explained first by the emergence of insecticide-resistant *Anopheles* species, which is currently considered one of the major challenges facing national malaria control programmes in Africa [27]. The same is true for DRC. For instance, a nationwide study conducted in several Congolese provinces showed a number of malaria vector species that were resistant to common insecticides used to treat bed nets [28]. In another study conducted in Kinshasa province where is located one of the sites of the present study, Riveron and colleagues found high resistance of *Anopheles gambiae* and *Anopheles funestus* to two commonly used insecticides, namely dichlorodiphenyltrichloroethane (DDT) and permethrin. This resistance phenomenon is worrisome as it leads to a reduced insecticidal effect of bed nets against *Anopheles* [29].

Additionally, in malaria hyperendemic zones such as Kinshasa and Kongo central provinces in DRC, the high mosquito population density, mainly due to poor WASH, contribute to increased malaria rates as people are exposed to mosquito bites almost anywhere before bedtime. A work by De Silva and Marshall [30] has shown that in the southern African countries, including DRC, the presence of mosquito breeding sites such as water puddles and seepages in homes and public areas was associated with malaria. This fact is in line with the findings of this study which suggest that poor WASH, particularly the use of inappropriate water source (which contribute to create water puddles) and latrines, and the absence of periodic sanitation intervention might have influenced household malaria frequency in our study sites.

The cross-sectional design is one of the limitations of this study, but nonetheless results may reflect the reality of the two study sites. Additionally, in this study, respondents had to report on malaria status in the previous 12-month period that preceded the survey, which may be subject to recall bias. Another fact is that, during hot weather in tropical regions, some individuals do not like to use ITN to protect themselves against mosquito bites. A study conducted by Guerra and colleagues [31] showed that, of the 40% of participants owning ITN in the Guinean village of Miyobo, only 36% ITN users has been consistently using this preventive measure. However, in this study, consistency in ITN use was not investigated.

## Conclusion

The present pilot study showed high malaria incidence in Congolese households in a context of extreme poverty, and household size, WASH status and rural site were revealed to be major factors associated with household malaria. To scale up malaria programme in DRC, anti-vector measures such as ITN and IRS should be made accessible to the populations at risk [32], along with the implementation of periodic WASH interventions in residential areas. This might possibly lead to an efficient control of malaria.

## Abbreviations

ACT: artemisinin-based combination therapy
DDT: dichlorodiphenyltrichloroethane
DRC: Democratic Republic of Congo
IRS: indoor residual spray
ITN: insecticide-treated net
LLIN: long-lasting insecticide-treated net
MDG: millennium development goal
MIQ: malaria indicator questionnaire
PHC: primary health care
RDT: rapid diagnostic test
SDG: sustainable development goal
SSA: sub-Saharan Africa
WASH: water, sanitation and hygiene
WHO: World Health Organization
